# A mutually-exclusive binary cross tagging framework for joint extraction of entities and relations

**DOI:** 10.1371/journal.pone.0260426

**Published:** 2022-01-21

**Authors:** Xuan Liu, Wanru Du, Xiaoyin Wang, Ruiqun Li, Pengcheng Sun, Xiaochuan Jing

**Affiliations:** 1 China Aerospace Academy of Systems Science and Engineering, Beijing, China; 2 Aerospace Hongka Intelligent Technology (Beijing) CO., LTD., Beijing, China; European Commission, ITALY

## Abstract

Joint extraction from unstructured text aims to extract relational triples composed of entity pairs and their relations. However, most existing works fail to process the overlapping issues that occur when the same entities are utilized to generate different relational triples in a sentence. In this work, we propose a mutually exclusive Binary Cross Tagging (BCT) scheme and develop the end-to-end BCT framework to jointly extract overlapping entities and triples. Each token of entities is assigned a mutually exclusive binary tag, and then these tags are cross-matched in all tag sequences to form triples. Our method is compared with other state-of-the-art models in two English public datasets and a large-scale Chinese dataset. Experiments show that our proposed framework achieves encouraging performance in F1 scores for the three datasets investigated. Further detailed analysis demonstrates that our method achieves strong performance overall with three overlapping patterns, especially when the overlapping problem becomes complex.

## Introduction

Relation extraction (RE) from natural language texts is widely studied in information extraction (IE). RE aims to detect specific types of entities from unstructured text and the semantic relations between entity pairs. It is the basis and data source for building knowledge bases (KBs) such as YAGO [1], Freebase [2], DBpedia [3] and NELL [4]. The relation is often formalized as a relational triple *T*, which consists of two entities (*E*1, *E*2) and the semantic relation *Rs* between them: *T* = <*E*1, *Rs*, *E*2>. For example: <Beijing, the capital of, China>.

Early works on RE adopted a pipeline approach [5, 6]. First, entity recognition is conducted using a named entity recognition (NER) module, then relations are further classified for each entity pair with a relation classification (RC) module [7]. Since errors in the early stage cannot be fixed in the following stage, such an approach suffers from propagation errors. Subsequent research introduced a joint learning method to ease error extraction. The joint extracting methods include feature-based models [8–11] and neural network models [12–15], and are able to extract and leverage the deep associations between entities and relations at the same time. However, in situations where sentences contain multiple overlapping triples, the effect of existing models fail to meet expectations.

**Fig 1:**
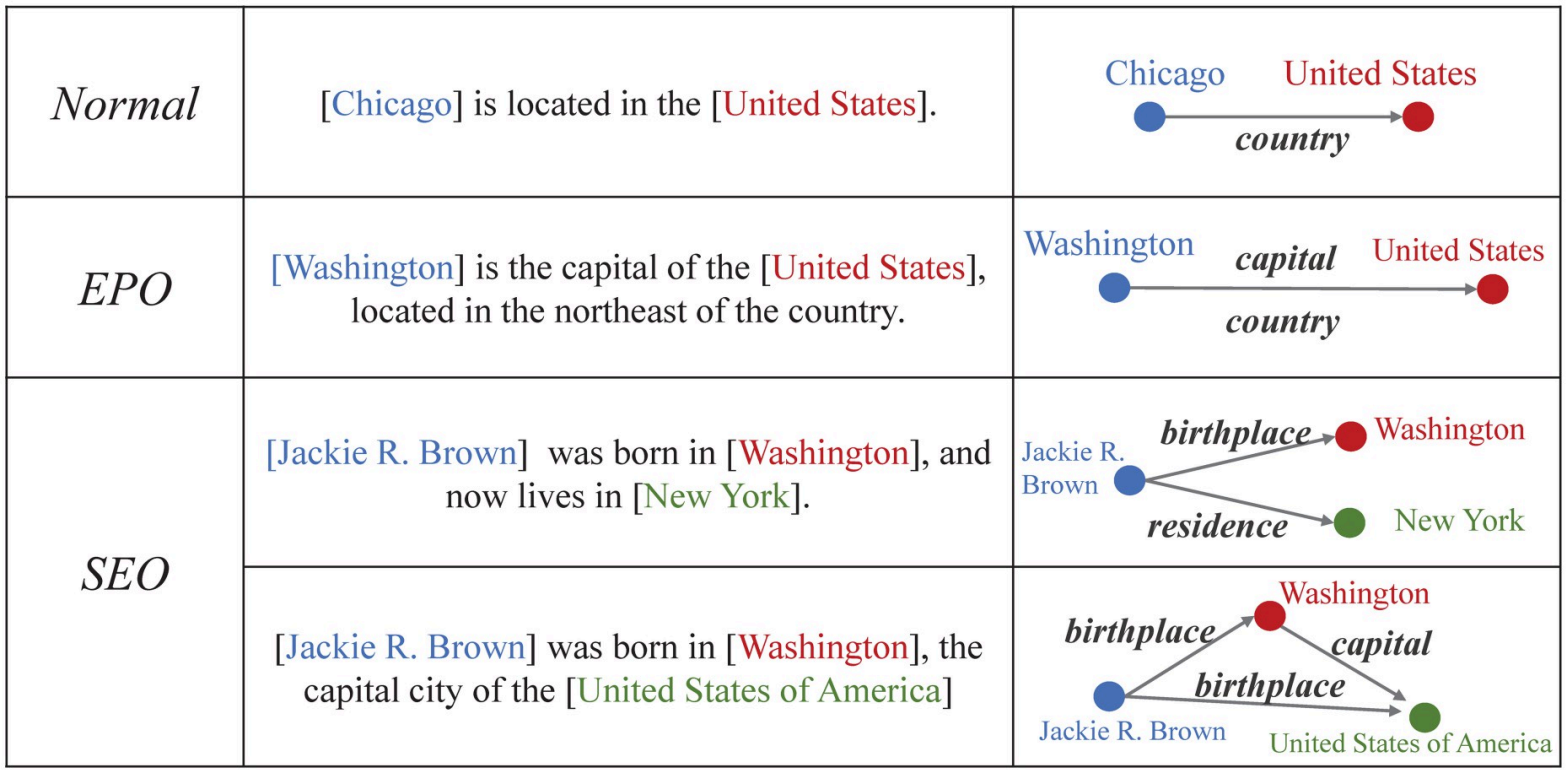
Examples of *Normal*, *EntityPairOverlap* (EPO) and *SingleEntityOverlap* (SEO) overlapping patterns.

The relational triples in the sentence have been divided into three types according to the overlap degree: *Normal*, *EntityPairOverlap* (EPO) and *SingleEntityOverlap* (SEO) [14] as shown in Fig 1. A sentence belongs to the EPO pattern if some of the triples have overlapping entity pairs. For instance, the two different relational triples <[Washington], capital, [United States]> and <[Washington], country, [United States]> share the same entity pairs. Alternatively, a sentence belongs to the SEO pattern if two triples contain at least one overlapping entity and do not share the same entity pairs. Consider the example sentence (as shown in Fig 1): “[Jackie R. Brown] was born in [Washington], and now lives in [New York].”. In this case, two different relational triples share the single entity [Jackie R. Brown]. In an even more sophisticated case, “[Jackie R. Brown] was born in [Washington], the capital city of the [United States of America].”, every single entity has an overlapping issue.

Subsequent works proposed some novel tagging schemes to handle the overlapping issues. Zeng et al. [14] introduced a sequence-to-sequence model with a copy mechanism to extract triples and to further investigate the impact of the extraction order [16]. Fu et al. [15] also built a Graph Convolutional Networks (GCNs) based model to study the overlapping triple problem. Nevertheless, these models predicted a single relation class for an entity pair and were not able to extract all the triples in a sentence. As a result, the overlapping triple is not addressed.

In our work, we aimed to design a framework that can extract entities and relations from Normal, EPO and SEO sentences while addressing the challenge mentioned above. Unlike previous tagging methods, we propose a novel mutually exclusive binary cross tagging scheme and developed the end-to-end BCT framework to improve the RE performance.

First, each token in a sentence is identified by the mutually exclusive binary tags, which represent the word position in the entity span. These binary tags are further distinguished by different predicates and subject/object dichotomy. Second, the binary tags of the two entities are cross-matched in all tag sequences to form the entity pairs that share the same relation type. If two or more triples have the same relation in an input sentence, the triples are formed based on the nearest principle. Finally, we establish a tagging scheme that can treat the task as token-level multi-label classification. Thus, a novel sequential tagging scheme representing the overlapping triple is used for relation extraction.

To enable our scheme, we propose an end-to-end framework for training that can perform joint extraction of relational triples from sentences following EPO and SEO patterns.

The key contributions are summarized as follows:

- We propose a mutually exclusive binary cross tagging scheme to label the overlapping triples in sentences of EPO and SEO types;
- We built an end-to-end framework based on our proposed tagging scheme to perform the joint extraction of overlapping triples in a sentence;
- Experiments on the three public datasets show that the end-to-end BCT framework achieves encouraging performance and consistent improvements in F1 score, obtained by effectively handling the overlapping issue through the mutually exclusive binary cross tagging scheme.

## Related work

Relation Extraction is a core task in Information Extraction and its goal is to extract relational triples from unstructured natural language text. It is critical for ontology learning works, particularly for building large-scale relational knowledge graphs [17]. In addition, it has been introduced to a variety of NLP applications such as text summarization and sentiment analysis.

Current IE research is mainly divided into fixed Information Extraction and Open Information Extraction (OIE). OIE was introduced as an open variant of the conventional IE task [18]. It focuses on extracting the text description of relations from plain texts without predefining a set of relationships. The first OIE system called Textrunner [19] utilizes a self-supervised learning framework to extract relational facts. Subsequent rule-based system ReVerb [20] employs handcrafted rules implemented by regular expressions to establish relations. However, both Textrunner and ReVerb only extract relational triples from the sentences connected to the verb. Later systems OLLIE [21] and ClausIE [22] were able to extract non-verbs as relation words using dependency-analysis algorithms. Even more recently, a study in never-ending learning called NELL [4] acquires knowledge through continuous reading and can reason its knowledge base.

Although the OIE task does not limit the relation classes, the extracted relationships tend to lack semantic information. Unlike the research in the OIE field, the fixed IE in this work utilizes pre-labeled datasets and predefined relationships for extracting semantic relationships. Despite the coverage rate being lower than OIE, the method is simple and easy to maintain in specific scenarios. Our work focuses on training an entity and relation extractor for predefined relations, intending to extract high-precision semantic relations for overlapping problems.

With the development of neural networks, researchers increasingly pay attention to fixed IE based on deep learning. More and more efforts are made to deal with relation extraction in complex scenarios. Early RE works [23, 24] mainly followed the pipelined method. The pipelined method extracts relational triples in two separate steps. First, the entities in a sequence are identified. Next, the relations between entities are distinguished by running relation classification (RC). Several pipeline methods based on neural network models have been proposed to improve the effectiveness. For example, Vu et al. [25] introduces Bidirectional RNN for the relation classification. Xu et al. [26] used LSTM for the RC module to obtain the information along the shortest dependency path. However, the accuracy of the RC module will be affected by preliminary errors using these pipeline methods. In addition, the pipeline method usually neglects the interaction between the two steps and creates unnecessary redundancy as a result. By introducing a tagging framework, we convert the relation extraction task into an end-to-end prediction problem where we simultaneously extract entities and relations, thereby alleviating the propagation error of the pipeline method.

Compared with pipeline methods, joint models can limit the error propagation by integrating entities and relations. Several joint models [8–11] have been proposed to detect entities and relations simultaneously, but these models rely on substantial manual work. Subsequently, Zheng et al. [13] proposed a novel tagging scheme for extracting multiple relations which converts the joint extracting models into a sequence tagging problem. However, this model can only assign one label to each word and cannot deal with overlapping triples, as the number of tags is too large to learn. Zeng et al. [14] classified the sentences with overlapping triples into EPO and SEO, and they used a Seq2Seq learning framework with a copy mechanism to ease the overlapping issues. Nevertheless, their NER module relies heavily on high-precision word segmentation tools. More recently, Fu et al. [15] proposed a novel joint method based on graph convolutional networks (GCNs). Luo et al. [27] employed two binary tree structures to solve the overlapping triple problem, but they would need to design a different handling approach to handle all three types of sentence modes. Despite their initial successes, none of these methods can fully extract the overlapping triples, and the aforementioned models fail to achieve satisfactory results when the problem of overlapping triples becomes relatively more complex. Our extraction framework is constructed based on the sequence tagging approach. We perform mutually exclusive binary labeling for each entity, then jointly extract entities and relations by a cross-match constraint. Subsequently, we adopt joint learning and utilize the end-to-end BCT framework to process the relational triples.

In this work, we propose a sequential tagging scheme and show how we efficiently employed an end-to-end neural model to extract relations without NER and RC. Our end-to-end BCT framework solves overlapping patterns with a sequential tagging scheme and reduces the number of tags to learn. Compared with the previous tagging schemes, our model achieves better results for sentences that include any of the types of overlapping triples.

## Methodology

**Fig 2:**
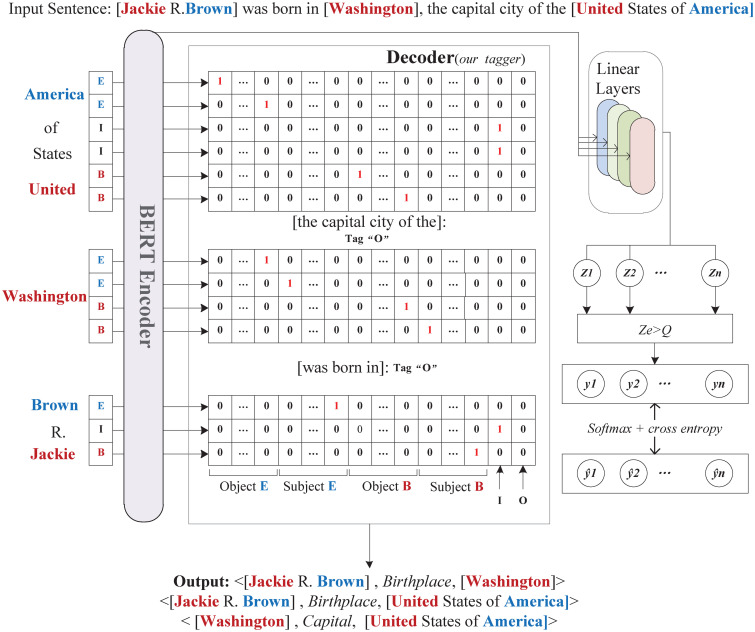
An illustration of our proposed end-to-end BCT extraction framework. In this example, red and blue respectively represent the “B” tags and “E” tags for the entities. We suppose there are |*N*| predicates, and a total number of 4|*N*| + 2 BCT tags, which are the 4|*N*| mutually exclusive “B” and “E” tags, plus two “I” and “O” tags.

Our proposed end-to-end BCT framework enables the extraction of multiple relational triples at once. In this section, we will introduce each part of the end-to-end BCT framework in detail. As Fig 2 shows, the proposed framework is composed of a BERT-based encoder, a decoder module (BCT tagger) and a loss function module. Our BCT scheme is mainly employed in the decoder part of the extraction framework, and we will illustrate in detail how to simplify the extraction problem to a concise tagging issue based on the BCT scheme later in this section.

### End-to-end extraction framework

Our proposed end-to-end BCT framework is illustrated in Fig 2. It contains the encoder module, decoder module, and loss function. The input of our model is a sentence *w* = [*e*_1_, *e*_2_, …, *e*_*n*_], where *e*_*t*_ represents the t-th word in the sentence of length *n*. The output semantic representation through the BERT-based encoder module is *Z*_*n*_. Next, we apply the BCT scheme to decode *Z*_*n*_ and lastly the entity and triples are jointly extracted.

#### BERT-based encoder

The encoder module aims to extract feature information for each word from the sentence *w*. In this paper, a pre-trained BERT model [28] is adopted as the encoder for input sentences. We will briefly introduce the BERT, a multi-layer bidirectional Transformer-based language representation model.

The pre-trained BERT model contains the Embedding module and *N* identical Transformers Block modules. The Transformer module uses multi-head attention to represent a word with a vector containing context information. We define the transformer module as *Trans*(*x*) where *x* represents the input vector. The transformer module extracts each word feature containing context information based on the multi-head attention. We can write the formula as follows:

$$\begin{array}{c} h=S*W_{s}+W_{p} \end{array}$$ (1)

$$\begin{array}{c} h_{a}=\text{T}rans\left(x\right),a\in \left[1,N\right] \end{array}$$ (2)

We first convert the input sentence into the matrix of one-hot vectors of sub-words to get the matrix *S* = [*x*_1_, *x*_2_, …, *x*_*n*_]. *S* is a matrix comprised of one-hot vectors of token indices in the input sentence. The sub-words embedding matrix is represented as *W*_*s*_ and the position embedding matrix is denoted as *W*_*p*_ where *p* represents the position index in the input sequences. The hidden state vector is represented as *h*, and *h*_*a*_ is the hidden state of the input sentence at the *a*-th layer. Since our input is a single sentence instead of a text pair, we did not consider segmentation embedding.

#### Decoder(BCT tagger)

Our BCT scheme is mainly employed in the decoder module. We first illustrate how to simplify the extraction problem to a concise tagging issue based on the BCT scheme and then introduce our decoding process.

**Fig 3:**
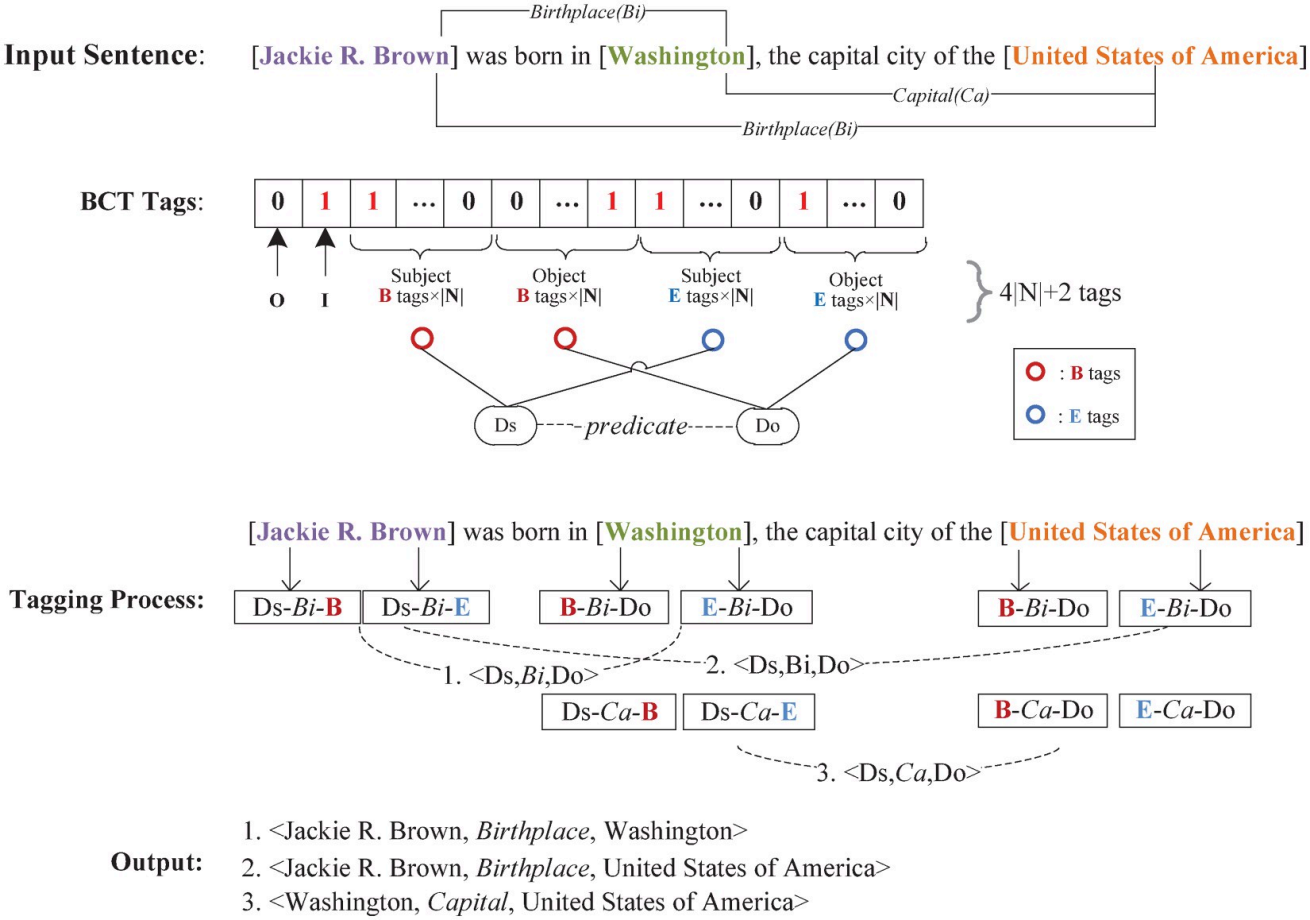
A visual illustration of our BCT scheme. In this example, red and blue circles represent respectively the “B” tags and “E” tags for the entities. We take an SEO sentence with 3 triples and 2 relation types as an example.

*Mutually exclusive binary cross tagging scheme*. Our mutually exclusive binary cross tagging scheme is designed to extract multiple overlapped EPOs and SPOs in the RE task. We use an example to detail our tagging strategy as shown in Fig 3, and the pseudo-code is presented in **Algorithm 1**.

Based on the conventional BIO tagging scheme [29], each token in the input sentence is assigned a label that contributes to indicate its position in an entity span. The conventional BIO tagging scheme [29] works based on the positions within a labeled entity. BIO refers to the beginning, inside, and outside of an entity. Likewise, we employ BIEO signs to label entities in our tagging scheme. The tags consist of three parts: the word position in the entity, the relation type, and the relation role.

- The word position in the entity: “BIEO”, where the signs B (begin), I (inside), E (end), and O (outside) represent the position information of a word in the entity.
- The relation type: The relation type information is obtained from a predefined set of predicates |*N*|.
- The relation role: The relation role information is represented by the subject *Ds* and the object *Do*. An extracted result is represented as a triple: <*Ds*, *predicate*, *Do*>. *Ds* means the subject entity in the triple, while *Do* belongs to the object entity.

Fig 3 illustrates an example of our tagging scheme. The input sentence “[Jackie R. Brown] was born in [Washington], the capital city of the [United States of America]” is a typical SEO overlapping pattern. It contains three overlapped triples: <Jackie R. Brown, Birthplace, Washington>, <Jackie R. Brown, Birthplace, United States of America> and <Washington, Capital, the United States of America>. Where “Birthplace” and “Capital” are the predefined relation types. The words “Jackie,” “R.,” “Brown,” “Washington,” “United,” “States,” “of,” and “America” are all related to the final extracted entities. Thus, we tag these words based on our BCT strategy.

Next, we generate BCT tags for each word in the input sentence. As Fig 2 shows, each token in a sentence is identified by the mutually exclusive binary “B” and “E” tags. These tags are further distinguished by different relations and subject/object dichotomy. A “B” or “E” tag should have the form of (B (E)-predicate-*Do*) or (*Ds*-predicate-B (E)). If we suppose there are |*N*| predicates, the results are 4|*N*| mutually exclusive “B” and “E” tags as shown in Fig 3.

In the example of our tagging process shown in Fig 3, the word “United” is the first word of the object entity “United States of America” and is related to the predicate “Capital”. The resulting triple based on the BCT tag then becomes (B-predicate-*Ds*). Similarly, the BCT tag for the last word of the object entity “America” is (E-predicate-*Ds*). Any words between “United” and “America” are now tagged as “I (Inside)” while the subject entity “Washington” (which is corresponding to “United States of America”) is tagged as (B-predicate-*Do*). Additionally, words unrelated to the final result are labeled as “O”.

The binary “B” and “E” tags of each entity are cross-matched with an interval of 2|*N*| in all tag sequences to form the subject or object. Matching tags that differ by |*N*| total tags will share the same predicate type. After tagging, we deal with the overlapping by using token-level multi-label classification, with a total of 4|*N*| labels plus the “I” and “O” tags. The decoder parses the word representations and predicts the BCT tags for each word. As shown in Fig 2, the *h*_*a*_ obtained from the BERT encoding layer is the input for the decoding layer. It is fed into linear layers to compute the probabilities of BCT tags based on the tag predicted vector.

The specific formula is as follows:

$$\begin{array}{c} Z_{k}=W_{k}*x_{i}+b_{k},\;k\in \left[B,I,E,O\right] \end{array}$$ (3)

$$\begin{array}{c} \begin{array}{c} p_{i}^{\text{k(B)}}=W_{B}x_{i}+b_{B}\\ p_{i}^{\text{k(E)}}=W_{E}x_{i}+b_{E} \end{array} \end{array}$$ (4)

Where the *x*_*i*_ is the i-th vector of *h*_*a*_, *W* represents the trainable weight and *b* is the bias. The encoding vector *Z*_*k*_ is the result of the output of the BERT and linear layers. The probabilities of recognizing the i-th token with “B” and “E” tag of an entity are indicated by respectively $p_{i}^{\text{k(B)}}$ and $p_{i}^{\text{k(E)}}$.

The essential decoding process of our BCT scheme is shown in **Algorithm 1**. The encoding vector *Z*_*k*_ generated by the BERT and linear layers is utilized as input of the decoder. Before the actual decoding process of joint extraction can start, we first have to perform some preparation and processing work to store the output of the encoder in a list *P*. No activation functions were used to implement our subsequent loss function. Instead, we require the probabilities of the target class to be higher than the other classes. It can be described as follows:

$$\begin{array}{c} d_{i}=\{\begin{array}{cc} Q & Z_{k}\in \left[-\infty ,0\right]\\ 0 & Z_{k}\in \left(0,+\infty \right] \end{array} \end{array}$$ (5)

$$\begin{array}{c} p(d_{i},Z_{k^{\prime }})=p(Z_{k^{\prime }}|d_{i})p(d_{i}) \end{array}$$ (6)

**Algorithm 1** Mutually-exclusive Binary Cross tagging scheme

**Input**: The predict results *Z*_*k*′_ for entities filtered by *Q* output by the BERT and linear layers.

**Output**: *Entity* list *E* and *SPO* list: *T* = <*E*1, *Rs*, *E*2>

1: Initialize list:*P* = [], entities list: *E* = [],*SPO* list: *T* = []

2: Sort elements of array *Z*_*k*′_ in *P* by position

3:**for** *i* in *length*(*P*) **do**

4:  Determine the initial position *i* of the entity;

5:  **if** *id* ∈ *P* **then**

6:   *id* is defined as the triple matching identifier; Determine the end position *j* of the entity, *j* = 0;

7:   **while** *i* + *j* + 1 < *length*(*P*) **do**

8:    **if** *id* + 2|*N*| in *P*[*i* + *j* + 1] **then**

9:     Add 1 to *j*;

10:     Form the entity with the start and the end index elements with an interval of 2|*N*| in *P*;

11:    **end if**

12:    **if** the *P*[*i* + *j* + 1] is the value of Inside sign (I = 1). **then**

13:     Add 1 to *j*

14:    **else if** *i* + *j* + 2 < *length*(*P*) **and** *P*[*i* + *j* + 2] is the value of Inside sign (I = 1). **then**

15:     Add 1 to *j*

16:    **else**

17:     Form the entity with the start and the end index elements with an interval of 2|*N*| in *P*;

18:    **end if**

19:   **end while**

20:  **end if**

21:  the “B” and “E” tags of two entities with an interval of |*N*| are cross-matched to form the triple;Add all entities to entities list *E*;Add the <*E*1, *Rs*, *E*2> to the *SPO* list: *T* = [];

22: **end for**

Here *Q* is a certain threshold. Eq (5) now helps us to filter for a certain target and form binary labels when *Q* = 1. A new set *Z*_*k*′_ is now generated under the constraint *d*_*i*_, and this new set *Z*_*k*′_ for entities filtered by *Q* is utilized as input for **Algorithm 1**. In this case, we sort elements of array *Z*_*k*′_ by position, and construct the list of the predicted results for entities *p*(*d*_*i*_, *Z*_*k*′_) (List *P* in **Algorithm 1**).

In the decoding process based on the BCT scheme, we declare three assistant extraction identifiers: *id*, *i*, and *j*. Here *i* and *j* are the location identifiers of an entity, and the id is the triple matching identifier. First, the start and end index elements that differ by 2|*N*| in the list are matched to form an entity (subject or object entity), then the binary tags of two entities with an interval of |*N*| are cross-matched to form a triple of the same predicate.

*BCT tags to triples*. In accordance to the tagged sequence in Fig 3, our BCT scheme can extract multiple triples at a time. As described above, we begin by extracting mutually exclusive dichotomous “B” and “E” tags for all subject and object entities. Then we start from the position of these “B” tagged entities and recursively match “E” tagged entities to form the subjects and objects. Finally, the “B” and “E” tags of two entities with an interval of |*N*| are cross-matched to form a triple of the same predicate, and we build the relations between the entities’ pair.

Whenever an input sentence has at least two triples with the same predicate, the triples are formed based on the nearest principle. Consider for example the following sentence: “The new station will have its headquarters in [Doha], [Qatar], and operate broadcast newsrooms in London, Washington and [Kuala Lumpur], [Malaysia]”. We know that “Doha”, “Qatar”, “Kuala Lumpur” and “Malaysia” share the same predicate “administrative-divisions”. Therefore, if a BCT tagged subject (“Doha” or “Kuala Lumpur”) can match more than one entity as its object while having more than one triple that shares the same predicate, we match the two entities that are closest in position in the sequence. In summary, we construct triples based on cross-matching with an interval of |*N*| and the nearest principle.

In this study, we do not add activation functions like the Sigmoid or Softmax function, but we employ the multi-label categorical cross-entropy to ease the class imbalance problems instead. The details are described below.

#### Loss function

The loss function for training our BCT tags is a multi-label categorical cross-entropy function. For the multi-label classification task, we need to select *m* target categories from *n* candidate categories. The common practice is to use the sigmoid activation function and then convert it to an |*N*| binary classification problem, with determination of the final loss through the Sigmoid cross-entropy. However, this approach will suffer from a severe category imbalance problem when *n* ≫ *m*.

As we know, the single-label classification task is more straightforward than multi-label classification; it can apply the efficient Softmax cross-entropy to avoid class imbalance. Multi-label classification on the other hand tends to create category imbalance problems. Therefore, we would like to extend the Softmax cross-entropy function to our task.

We employ a pair similarity optimization viewpoint [30], aiming to maximize the within-class similarity and minimize the between-class similarity. Given a single sample *x* in the feature space, we assume that *K* within-class similarity scores and *L* between-class similarity scores are associated with *x*. Since each target-class score *s*_*j*_ needs to be higher than non-target class score *s*_*i*_, we employ *logSumExp* as follows:

$$\begin{array}{c} \text{log}\;\text{SumExp}(x_{1}\ldots x_{n})=\text{log}(1+\sum_{i\in K,j\in L}e^{s_{i}-s_{j}}) \end{array}$$ (7)

The *K*, *L* sets are also the category sets of respectively negative and positive samples. In this study, *m* target categories are selected from *n* candidate categories, and we define a threshold *Q* = 0 as presented in **Algorithm 1** to control the output when *m* is not fixed. The detailed operations are as follows:

$$\begin{array}{ll} L_{m} & =\text{log}\;\left(1+\sum_{i\in K,j\in L}e^{s_{i}-s_{j}}+\sum_{i\in K}e^{s_{i}-Q}+\sum_{j\in L}e^{Q-s_{j}}\right)\\ & =\text{log}\;\left(e^{Q}+\sum_{i\in K}e^{s_{i}}\right)+\text{log}\;\left(e^{-Q}+\sum_{j\in L}e^{-s_{j}}\right) \end{array}$$ (8)

Where *L*_*m*_ is the multi-label categorical cross-entropy function including both the positive and negative samples. We derive *L*_*pos*_ for the positive labels and *L*_*neg*_ for the negative labels by Eq (8). The total loss *L*_*m*_ of our framework is then determined as follows:

$$\begin{array}{ll} L_{m} & =\text{log}\;\left(1+\sum_{i\in K}e^{s_{i}}\right)+\text{log}\;\left(1+\sum_{j\in L}e^{-s_{j}}\right)\\ & =L_{neg}+L_{pos} \end{array}$$ (9)

## Experiments and results

### Experimental setting

#### Datasets and evaluation metrics

The framework in this study has been evaluated on three widely used public datasets: NYT [31], WebNLG [32], and DuIE [33]. The NYT dataset was initially generated by the distant supervision method. It contains 1.18 million sentences with 24 predefined relations from New York Times articles. After removing sentences without valid triples, 61,195 sentences remain. We used the NYT datasets released by Zeng et al. [14], which includes a training set of 56,195 sentences, a validation set of 5000 sentences and a testing set of 5000 sentences. The WebNLG dataset was created for Natural Language Generation (NLG) tasks. We use the WebNLG datasets processed by Zeng et al. [14], which contains 246 predefined relation types. DuIE is a large-scale dataset built by Baidu Inc for Chinese information extraction, consisting of 210,000 sentences and 450,000 instances covering 49 predefined relation categories. We used the training set and development set from the DuIE dataset, which contains 173,108 sentences for training and 21,639 sentences for testing.

Table 1 describes the statistics of the training and testing sets from the three datasets. For each of the datasets, we divided the sentences into three different overlapping patterns of relational triples: Normal, *EntityPairOverlap* (EPO), and *SingleEntityOverlap* (SEO). Although several sentences might belong to both the EPO and SEO classes, the overlapping problem is common in these datasets.

**Table 1:** Statistics of NYT, WebNLG and DuIE datasets in our experiment.

Category	NYT		WebNLG		DuIE	
	Train	Test	Train	Test	Train	Test
Normal	37013	3266	1596	246	75600	9404
EPO	9782	978	227	26	7518	922
SEO	14735	1297	3406	457	95042	11957
ALL	56195	5000	5019	703	173108	21639

We follow the evaluation metrics from Fu et al. [15], that is to say that a predicated relational triple *T* = <*E*1, *Rs*, *E*2> is considered as a correct one only if the entities (*E*1, *E*2) and relation Rs are all correct. Specifically, we adopt the standard Precision (*Prec*.), Recall (*Rec*.) and F1 score (*F*1) to evaluate our framework.

#### Baseline methods

We selected several classical triple extraction models as our baselines. A list of the models can be found in Table 2. NovelTagging [13] uses the neural network to jointly extract relational triples by a novel sequential tagging scheme. CopyRE [14] is the first framework to consider the relational triple overlap problem. It proposed an end-to-end model based on sequence-to-sequence learning with a copy mechanism for relation extraction. CopyRE_*RL*_ [16] is a reinforcement learning method based on a sequence-to-sequence model to handle the multiple relation extraction tasks. Graph_*Rel*_ [15] is an end-to-end joint extraction model based on GCNs. BiTT [27] is an end-to-end extraction framework that labels the overlapping triples in a sentence based on two binary tree structures. CasRel [34] is a novel cascade binary tagging framework that is considered the state-of-the-art when it comes to results for the NYT and WebNLG datasets. CasRel regards the relations as mapping functions from subjects to objects. Note that in Table 2, CopyRE _*OneDecoder*_ and CopyRE _*MultiDecoder*_ are the CopyRE frameworks with one decoder and multiple decoders respectively. GraphRel_1*p*_ is the 1st phase for GraphRel architecture and GraphRel_2*p*_ is the complete version. We use these baseline models for further comparison, which have been upgraded through the pre-trained BERT [27].

**Table 2:** Results of different methods on NYT, WebNLG and DuIE datasets.

Method	NYT			WebNLG			DuIE		
	*Prec*.	*Rec*.	*F*1	*Prec*.	*Rec*.	*F*1	*Prec*.	*Rec*.	*F*1
NovelTagging [13]	62.4	31.7	42.0	52.5	19.3	28.3	-	-	-
NovelTagging _*BERT*_ [13]	89.0	55.6	69.3	-	-	-	75.0	38.0	50.4
CopyRE _*OneDecoder*_ [14]	59.4	53.1	56.0	32.2	28.9	30.5	-	-	-
CopyRE _*MultiDecoder*_ [14]	61.0	56.6	58.7	37.7	36.4	37.1	-	-	-
CopyRE _*RL*_ [16]	77.9	67.2	72.1	63.3	59.9	61.6	-	-	-
GraphRel _1*p*_ [15]	62.9	57.3	60.0	42.3	39.2	40.7	52.2	23.9	32.8
GraphRel _2*p*_ [15]	63.9	60.0	61.9	44.7	41.1	42.9	41.1	25.8	31.8
CasRel [34]	89.7	89.5	89.6	93.4	90.1	91.8	-	-	-
BiTT_*BERT*_ [27]	89.7	88.0	88.9	-	-	-	75.7	80.6	78.0
BCT_*BERT*_	**89.8**	**88.5**	**89.1**	**90.6**	**92.0**	**91.3**	**78.4**	**82.6**	**80.4**

We combined our proposed BCT scheme and the pre-trained BERT model to optimize performance. BCT_*BERT*_ is the full-fledged framework using pre-trained BERT weights.

#### Implementation details

In this work, we employ two types of pre-trained BERT models for fine-tuning: The BERT-Base uncased model and the BERT-Base Chinese model [28].

The BERT-Base uncased model (12-layer Transformer, 768-hidden, 12-heads, 110M parameters) is trained on a large text corpus (Wikipedia and BookCorpus) [28], and it is applied for fine-tuning two English datasets NYT and WebNLG. Furthermore, it employs Word Piece tokenization to split words.

However, this approach is not appropriate for Chinese datasets, as Chinese is a continuous language, where contrary to English, whitespace characters do not exist. Secondly, Chinese characters are the smallest unit and cannot be further split. For these reasons, we employ the BERT-Base Chinese model (12-layer Transformer, 768-hidden, 12-heads, 110M parameters) [28] to the DuIE Chinese dataset. This model utilizes character-based tokenization and is trained on the relevant corpus of Chinese Wikipedia.

In our experiments, we adopted a mini-batch mechanism with a batch size of 4 to train our model. The learning rate was set to 1*e*^−5^. Additionally, we implemented the early-stopping mechanism to prevent our model from over-fitting. Specifically, the training process is terminated when the F1 scores on the validation set do not increase for at least ten sequential epochs. These hyperparameters were determined by the validation set.

### Experimental results

#### Compared results

In this section, we compare our proposed method with the previously mentioned state-of-the-art models. We conducted experiments on all types of sentences (Normal, EPO and SEO) and compared the performance with results from previous works. Table 2 shows the results (*Prec*., *Rec*., and *F*1) of different baseline models, together with our framework for three datasets.

Our BCT_*BERT*_ framework achieves encouraging F1 scores in NYT, WebNLG, and DuIE datasets of 89.1%, 91.3%, and 80.4% respectively. Compared with the best current baseline method BiTT_*BERT*_ [27] for the NYT dataset, our BCT based on pre-trained BERT achieves strong performance, specifically in terms of F1. Notably, the results of the WebNLG dataset even outperform the NYT results since the proportion of overlapping triples is higher in the former. Our framework is comparable to the current state-of-the-art method CasRel [34] on the WebNLG dataset in F1 score. For the DuIE dataset, our framework outperforms the top baseline method BiTT_*BERT*_ [27] by 2.7% in precision, 2.0% in recall, and 2.4% in F1 score. From these results, we note that the BCT scheme helps to predict relations in terms of precision, recall, and F1 score in the general case.

NovelTagging [13], CopyRE _*MultiDecoder*_ [14], and our framework all use a sequential framework. NovelTagging considers all entities belonging to a single relation type, resulting in high precision and low recall. CopyRE _*MultiDecoder*_ applies multiple separated decoders to form relation triples, but the accuracy of extraction is low as a result of the restrictions on the copy mechanism. Our method, on the other hand, distinguishes the entities by different relations and subject/object dichotomy to generate more relation triples. This enables us to achieve high precision and high recall, yielding higher *F*1 scores.

A significant difference can be noted between the performance scores of NYT, WebNLG, and DuIE datasets in Table 2. The explanation for this lies in the differences in composition of specific datasets. The proportion of overlapping pattern sentences is relatively high in the WebNLG and DuIE datasets, while the NYT dataset mainly consists of Normal pattern sentences. Furthermore, the DuIE is a large-scale dataset with a larger proportion of EPO and SEO sentences. For most baseline methods these differences between the datasets generally cause the results for the NYT and WebNLG datasets to be better than the results for the DuIE dataset. However, our BCT model achieves competitive results on all three datasets, verifying the utility of the BCT scheme in solving overlapping triples.

#### Detailed analysis on different types of sentences

To further evaluate the capacity of the proposed end-to-end BCT framework in extracting overlapping relational triples, we experimented with different overlapping patterns and compared the results with prior works.

**Fig 4:**
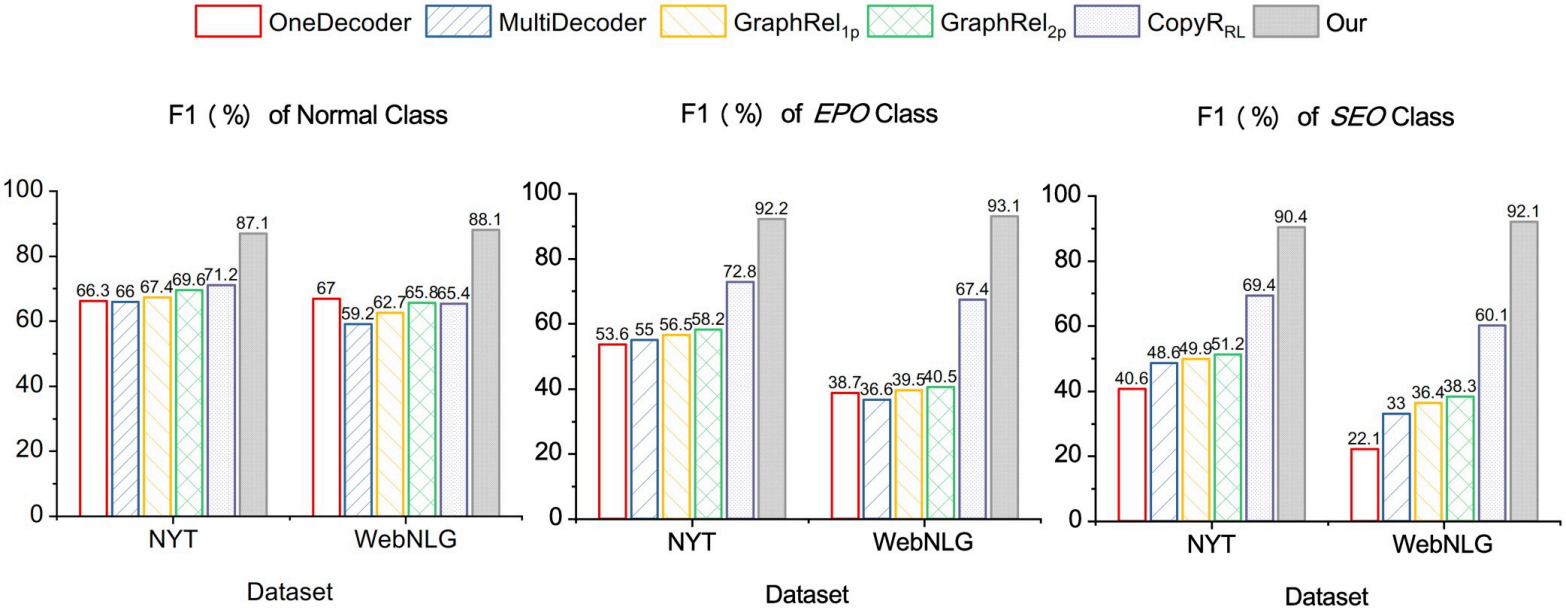
Results(F1-scores) of extracting relational triples from sentences with *Normal*,*EPO*, and *SEO* overlapping patterns.

Fig 4 shows the detailed results for both NYT and WebNLG datasets for three different overlapping patterns. As can be seen, our framework achieves much higher performance on all three overlapping patterns. Since the overlapping patterns make it harder to extract relational triples from sentences, the extracting performance of EPO and SEO patterns is significantly lower than the Normal pattern extraction performance in baseline methods. Contrarily, our framework shows consistently strong overall performance with all three overlapping patterns. This is because the BCT scheme was specifically designed to be more suitable for overlapping relational triples.

Additionally, we validated the ability to extract relational triples from sentences with different numbers of triples. Table 3 presents our method in detail. The sentences from the NYT and WebNLG test set have been split into five subclasses based on the number of triples. Our framework achieves superior overall performance in all five subclasses.

**Table 3:** F1-score of extracting relational triples from sentences that contain different numbers of triples.

Method	NYT					WebNLG				
	*N* = 1	*N* = 2	*N* = 3	*N* = 4	*N* ≥ 5	*N* = 1	*N* = 2	*N* = 3	*N* = 4	*N* ≥ 5
CopyRE _*OneDecoder*_ [14]	66.6	52.6	49.7	48.7	20.3	65.2	33.0	22.2	14.2	13.2
CopyRE _*MultiDecoder*_ [14]	67.1	58.6	52.0	53.6	30.0	59.2	42.5	31.7	24.2	30.0
GraphRel _1*p*_ [15]	69.1	59.5	54.4	53.9	37.5	63.8	46.3	34.7	30.8	29.4
GraphRel _2*p*_ [15]	71.0	61.5	57.4	55.1	41.1	66.0	48.3	37.0	32.1	32.1
CopyRE _*RL*_ [16]	71.7	72.6	72.5	77.9	45.9	63.4	62.2	64.4	57.2	55.7
CasRel [34]	88.2	90.3	91.9	94.2	83.7	89.3	90.8	94.2	92.4	90.9
BCT_*BERT*_	**87.3**	**90.5**	**88.7**	**94.4**	**87.3**	**87.8**	**90.4**	**94.6**	**93.0**	**91.2**

We divide the sentences of the NYT and WebNLG test set into 5 subclasses respectively. Each class contains sentences that have 1,2,3,4 or ≥ 5 triples.

**Fig 5:**
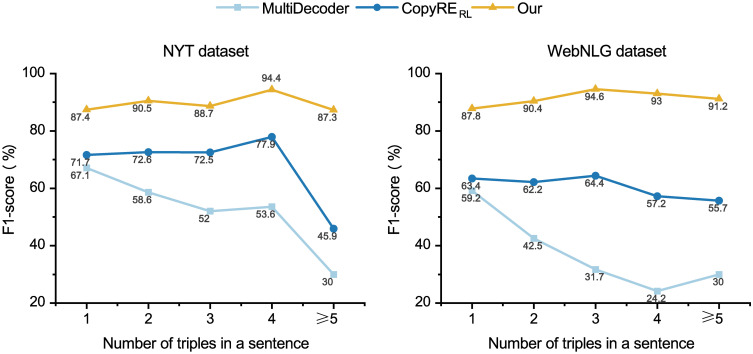
Performance(F1-scores) of extracting relational triples from sentences that contain different numbers of triples.

The results compared to the sequence-to-sequence learning methods are shown in Fig 5. It can be seen that the F1 scores of the two sequential methods display a downward trend with the increasing number of relational triples. Our framework gives a consistently stable performance on both of the investigated datasets. Especially when it becomes difficult to extract triples (the number of triples in a sentence ≥ 5), our framework gains the most significant improvement of F1 score over the other methods. Since the BCT scheme considers the various word features of entities based on mutually exclusive cross dichotomy, our method is superior in dealing with overlapping triples, resulting in a higher F1 score.

The experimental results on three public datasets show the effectiveness of our proposal, especially while dealing with overlapping entities and triples. Even so, still some shortcomings in the extraction of overlapping relations remain. Our BCT model focuses on extracting as many relational triples as possible from an input sentence, while the tagging process of entities considers all relation types simultaneously. Due to this characteristic, our framework is prone to mistakenly detect redundant triples when the number of triples in a sentence increases. Therefore, the relation identification between entity pairs needs to be further refined in subsequent work.

#### Case study

We conducted the case study by comparing our framework with the current best state-of-art method. Table 4 shows the results of different overlapping sentences containing Normal, EPO, and SEO patterns. It can be observed that both our framework and the CasRel [34] method present a solid ability to extract overlapping relational triples on the Normal and EPO sentences. However, CasRel could not accurately extract all the gold triples when dealing with a more complex SEO sentence. It failed to catch the triple < Brooklyn, contains, Island >, possibly due to this method’s specific general bias caused by relations being modeled as functions. Our method accurately captured all the overlapping relational triples, showing the effectiveness of the BCT scheme for dealing with overlapping cases. Although our framework achieved encouraging results in extracting overlapping triples, the process still requires refinement. We will continue to conduct research, with the goal of further improving our ability to reliably extract triples.

**Table 4:** Case study of our BCT model and best baseline method.

Sentence	Gold triples	CasRel [34]	Our
Kalle Palander of Finland won the race, and Akira *Sasaki* of *Japan* was the runner-up.	<*Sasaki*,nationality,*Japan*>	<*Sasaki*,nationality,*Japan*>	<*Sasaki*,nationality,*Japan*>
The issue reflects the relentless battle over *Jerusalem*, which both *Israel* and the Palestinians claim as their capital.	<*Israel*,capital,*Jerusalem*> <*Israel*,contains,*Jerusalem*>	<*Israel*,capital,*Jerusalem*> <*Israel*,contains,*Jerusalem*>	<*Israel*,capital,*Jerusalem*> <*Israel*,contains,*Jerusalem*>
He trained for about six months, he said, running from his house on East 23rd Street in *Midwood*, *Brooklyn*, to the Coney *Island* boardwalk and back, he said.	<*Brooklyn*,contains,*Midwood*> <*Island*,neighborhood of,*Brooklyn*> <*Brooklyn*,contains,*Island*> <*Midwood*,neighborhood of,*Brooklyn*>	<*Brooklyn*,contains,*Midwood*> <*Island*,neighborhood of,*Brooklyn*> <*Midwood*,neighborhood of,*Brooklyn*>	<*Brooklyn*,contains,*Midwood*> <*Brooklyn*,contains,*Island*> <*Midwood*,neighborhood of,*Brooklyn*) <*Island*,neighborhood of,*Brooklyn*>

Gold triples: All triples we expect to be extracted from the example sentences.

## Conclusions

In this work, we introduced an end-to-end BCT framework to jointly extract the overlapping entities and relations. Unlike previous sequential frameworks, we utilize an efficient binary cross-matching method for constructing entities that participate in multiple triples. The experimental results on three datasets show the effectiveness of our proposal, especially while handling overlapping issues. However, some shortcomings in the extraction of overlapping relations still remain. Our BCT model focuses on extracting as many relational triples as possible from an input sentence, while the tagging process of entities considers all relation types simultaneously. Due to this characteristic, our framework is inclined to detect redundant triples by mistake when the number of triples in a sentence increases. Therefore, the identification of relations between entity pairs needs refinement in further research. Our later work will be aimed at both enhancing our capacity to extract overlapping relational triples and applying our improved method to other tasks such as Medical Named Entity Recognition and Chinese event extraction.
